# Supplementary material for: Over-elongation of centrioles in cancer promotes centriole amplification and chromosome missegregation
Source: Nat Commun. 2018 Mar 28;9:1258. doi: 10.1038/s41467-018-03641-x (PMC5871873; doi:10.1038/s41467-018-03641-x)
Supplement: Supplementary file 1 — Supplementary Information(PDF 2137 kb) [file 41467_2018_3641_MOESM1_ESM.pdf]

Over-elongation of Centrioles in Cancer Promotes Centriole Amplification and Chromosome Missegregation

Marteil *et al*

SUPPLEMENTARY INFORMATION

a

b

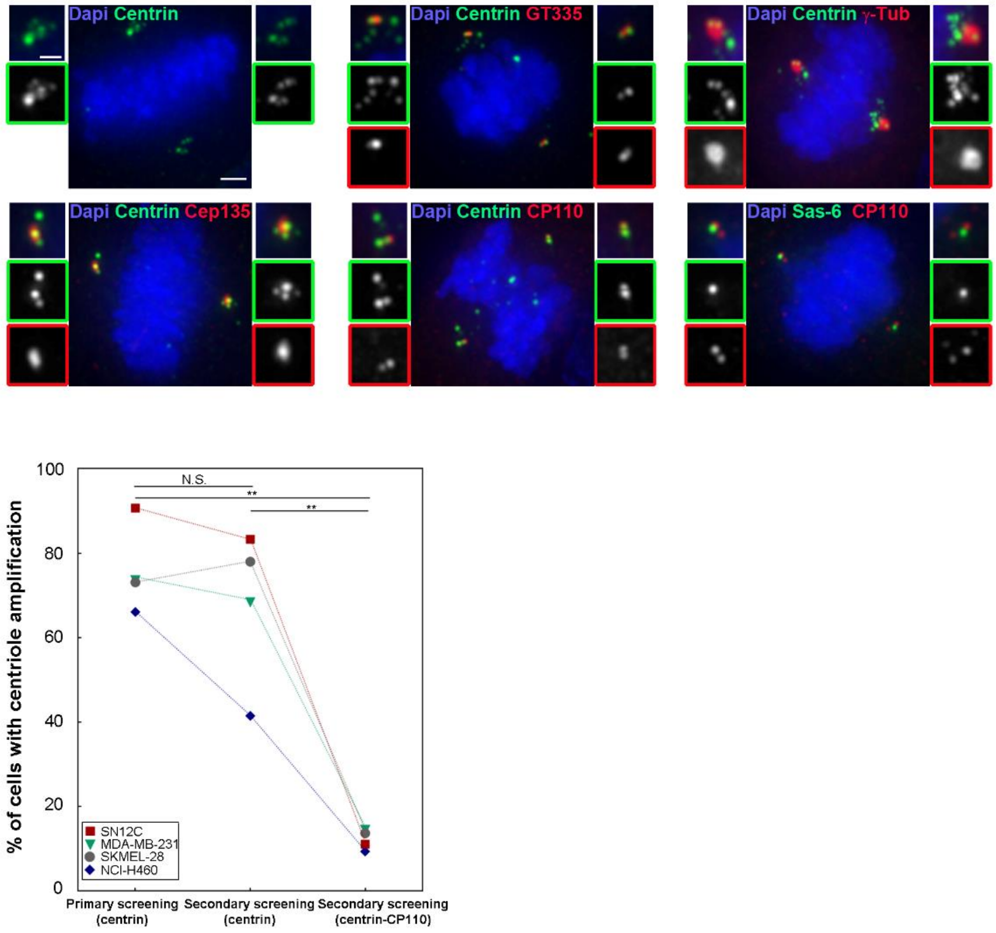

Supplementary Figure 1. a Centrin-CP110 is the best combination of *bona fide* markers for centriolar identification and length assessment in the secondary screening. Immunofluorescence pictures of SN12C cells stained with different combinations of centriolar-centrosomal antibodies: centrin (green)/GT335 (red), centrin (green)/α-tubulin (red), centrin (green)/Cep135 (red), centrin (green)/CP110 (red) and Sas-6 (green)/CP110 (red). Supernumerary centrin dots (>2) are often visible at each pole, but they rarely co-localise with the second marker. This suggests that these dots are not *bona fide* centrioles, therefore reinforcing the need of a second marker to correctly identify centrioles. CP110 was chosen since it is the only marker that gives a clear and single dot for each centriole. Scale bar 2 µm, insets 1 µm. **b** The presence of CP110-negative centrin *foci* is responsible for the observed discrepancies in the percentage of cells with centriole amplification between the primary and the secondary screenings. The differences observed between the two screenings could reflect (i) a lack of samples and/or centrin counts reproducibility or ii) the fact that centrin labels satellites besides *bona fide* centrioles or, iii) the presence of centrioles lacking CP110. To test this, we analysed the two datasets in the same conditions, i.e. using only centrin to quantitate centrioles. To do this, we first selected the four cell lines displaying the highest discrepancies between the primary and the secondary screenings, namely NCI-H460 (blue), MDA-MB-231 (green), SKMEL-28 (grey) and SN12C (red). We then re-quantified the percentage of cells with amplification in the secondary screening using only centrin as a centriolar marker. As shown in the graph, while the percentages of cells with amplification in the secondary screening are low when centrin is used in combination with CP110, they are very high and not different from to the ones observed in the primary screening when only centrin was used (paired t test, N.S.: Not significant and ** represents $p \leq 0.01$). These results demonstrate that the observed discrepancies in the percentage of cells with centriole amplification between the primary and the secondary screenings are likely due to the fact that centrin labels other structures than *bona fide* centrioles as suggested before[1].

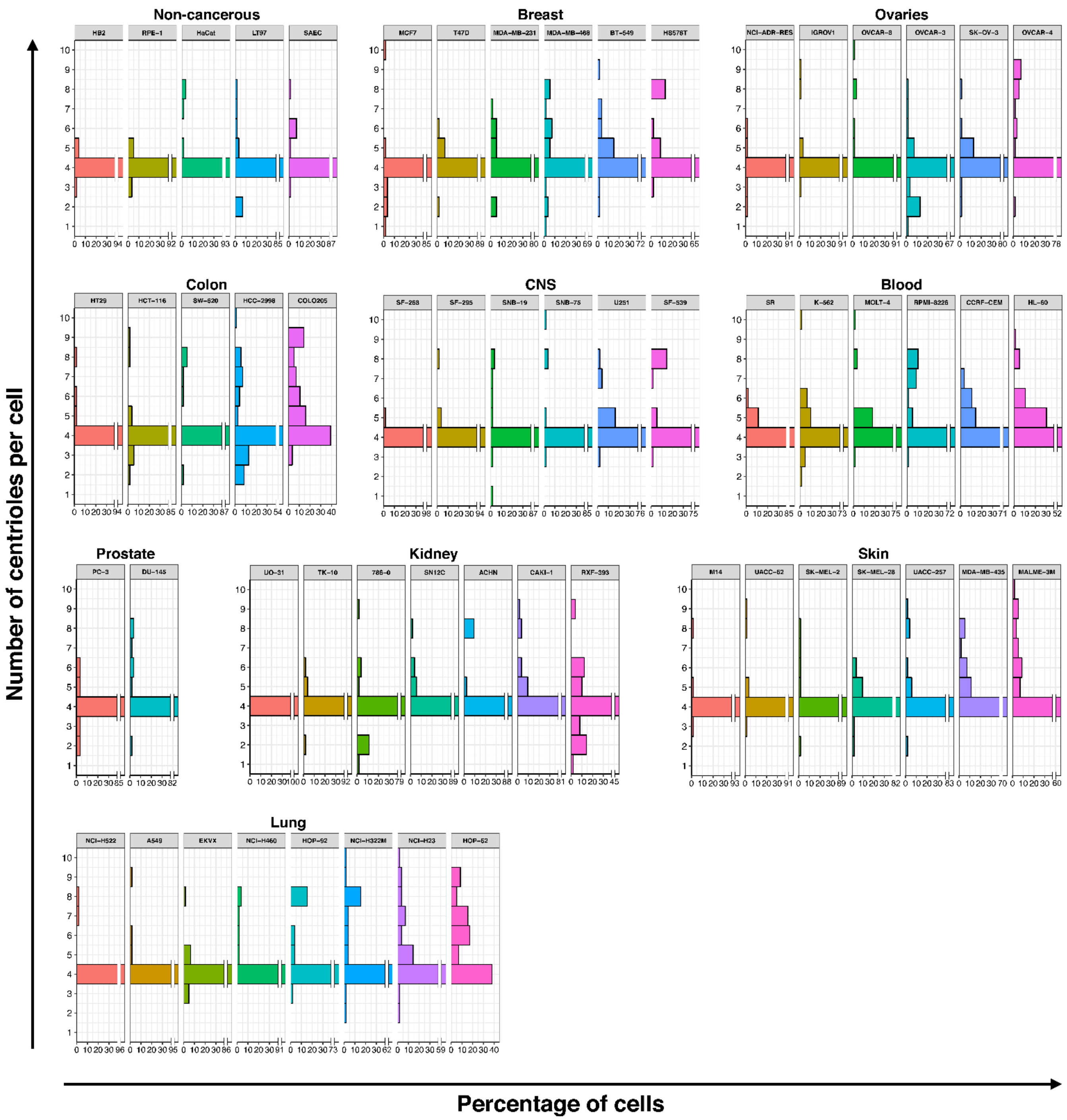

Supplementary Figure 2. Distribution of the number of centrioles per cell across the different cell lines of the NCI-60 panel, organised per tissue of origin, and across the 5 non-cancerous cell lines. Cell lines were ranked within each tissue by increased percentage of cells with >4 centrioles. Only percentages for cells with one to ten centrioles are shown but they were calculated based on all the cells for each cell line.

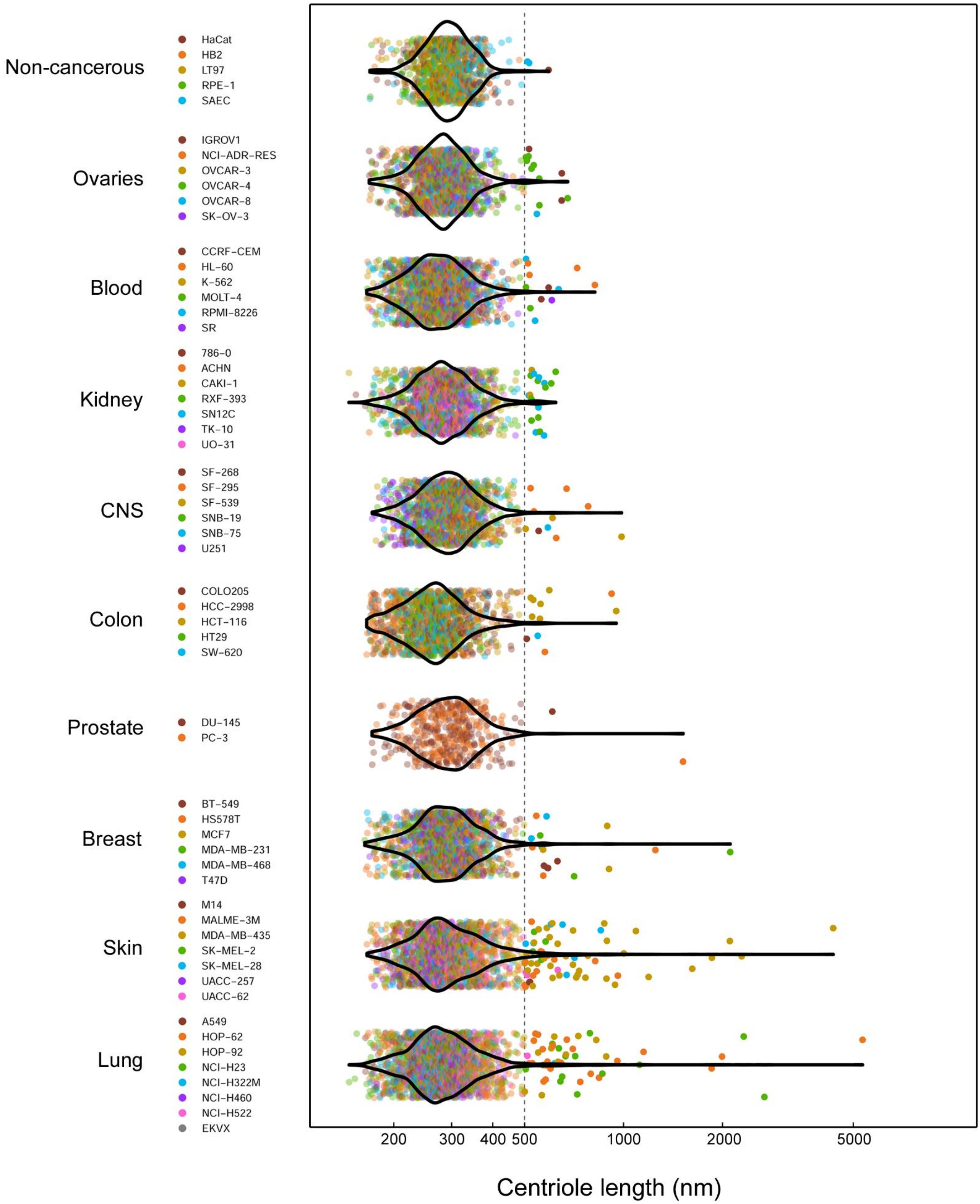

Supplementary Figure 3. Distribution of centriole length per tissue of origin of the NCI-60 panel. Length of individual centrioles (nm) across the 5 non-cancerous cell lines and across the NCI-60 tissues of origin, coloured by cancer cell line. Tissues are ordered by the variance of the respective distribution. The dotted line at 500 nm represents the cut-off used to consider a centriole as overly-long (i.e. twice the length of a normal-length centriole measured using centrin staining). The Y axis is in logarithmic scale.

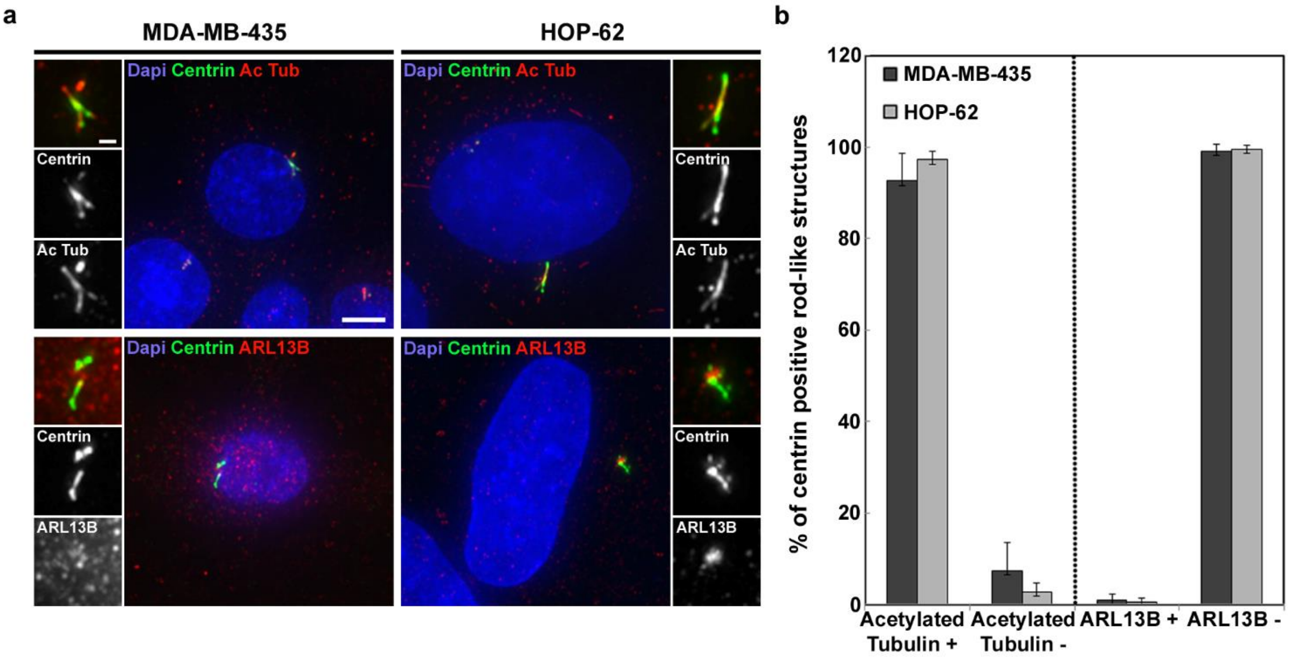

Supplementary Figure 4: Centrin-positive rod-like structures are *bona fide* centrioles and not cilia. **a** Representative immunofluorescence images of two cell lines, HOP-62 and MDA-MB-435, displaying centriole length deregulation and stained with centrin antibody (green) and acetylated-tubulin (stable MT marker of centrioles and cilia) or ARL13B (cilia marker) antibodies (red). DNA was stained using DAPI. Cells were subjected to a 2 h cold treatment, prior to fixation, to depolymerise the cytoplasmic MTs. Scale bar 5µm, insets 1 µm. **b** Bar graph showing the percentage of centrin rod-like structures positive or negative for either acetylated-tubulin or ARL13B for MDA-MB-435 and HOP-62 cell lines. Please note that nearly all the centrin-positive structures in both cell lines were positive for acetylated tubulin (93 +/- 6% for MDA-MB-435 and 97 +/- 2% for HOP-62) and negative for ARL13B (99 +/- 2% MDA-MB-435 and 100 +/- 1% for HOP-62, therefore confirming their centriolar nature. Data represent the mean +/- s.d. of three independent experiments (at least 30 centrioles were analysed per condition/ per experiment).

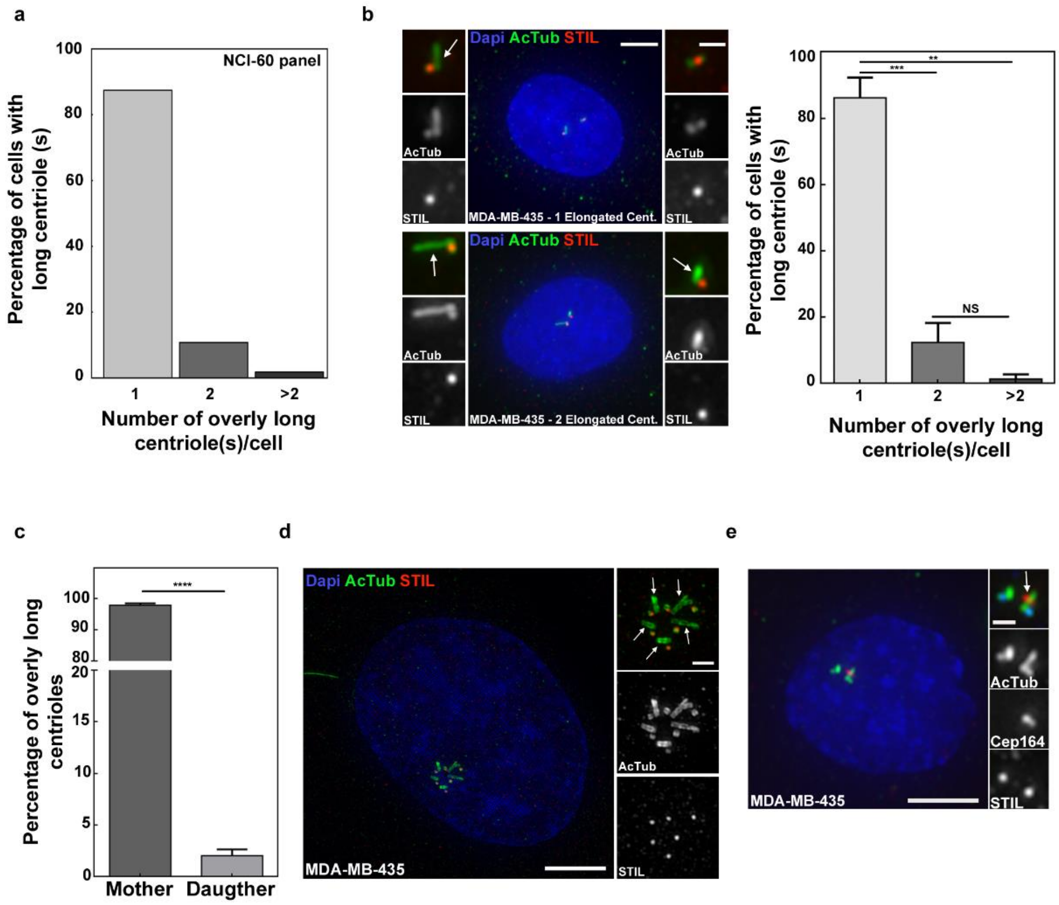

Supplementary Figure 5: Centriole length deregulation does not affect all centrioles within each cell. **a** Distribution of the number of overly long centrioles in each cell from the NCI-60 secondary screening. Most cells with centriole length abnormalities only have one centriole affected by over-elongation, as expected from a rare event distribution. **b** Validation of the screening results: most of MDA-MB-435 cells with centriole length deregulation display only one elongated centriole. Immunofluorescence images of MDA-MB-435 cells displaying one (upper panel) or two (lower panel) elongated centriole(s) (labelled with white arrows). Cells were subjected to a 2 h cold treatment, prior to fixation, to depolymerise the cytoplasmic MTs and were stained with DAPI (blue), acetylated-tubulin (green) and STIL (red) antibodies. Data represent the mean + s.d. of three independent experiments (n=68,70 and 73). *** $p \leq 0.001$, ** $p \leq 0.01$, NS not significant (unpaired t test with Welch's correction). **c and d** Centriole over-elongation affects the older/mother centrioles in MDA-MB-435 cell line. **c** Bar graph showing the percentage of overly long centrioles that are either mother (negative for procentriolar marker, STIL, staining) or daughter (positive for STIL staining) centrioles. This quantification was performed in the same time as **b** quantification. Data represent the mean + s.d. of three independent experiments (n=83,76 and 85). **** $p \leq 0.0001$ (Unpaired t test with Welch's correction). **d** Structured Illumination Microscopy (SIM) of MDA-MB-435 cells validating that centriole over-elongation affects mother centrioles (same experimental details as in **b**). Note that overly long centrioles (labelled with white arrows) are lacking STIL staining. **e** Centriole over-elongation likely affects mainly grand-mother centrioles in MDA-MB-435 cell line. Immunofluorescence image of a S-G2 phase MDA-MB-435 cell showing that the elongated centriole (labelled with a white arrow) is the one positive for the distal appendages protein, CEP164, therefore being the grand-mother centriole. Cells were subjected to a 2 h cold treatment, prior to fixation, to depolymerise the cytoplasmic MTs and subsequently stained with DAPI (blue), and acetylated-tubulin (green), CEP164 (red) and STIL (blue in the insets) antibodies. For all images, scale bar: 5 µm. Insets: 1 µm.

a

			Number of centrioles per cell			Total number of cells counted	Number of patients counted	Range of cells counted per patient
			≤ 4	5 to 8	>8			
Molecular Subtype	Luminal	Number of cells	602	52	1	655	15	27-133
		% of cells	91,9	7,9	0,2	100		
	Basal	Number of cells	572	159	3	734	10	20-107
		% of cells	77,9	21,7	0,4	100		

b **c**

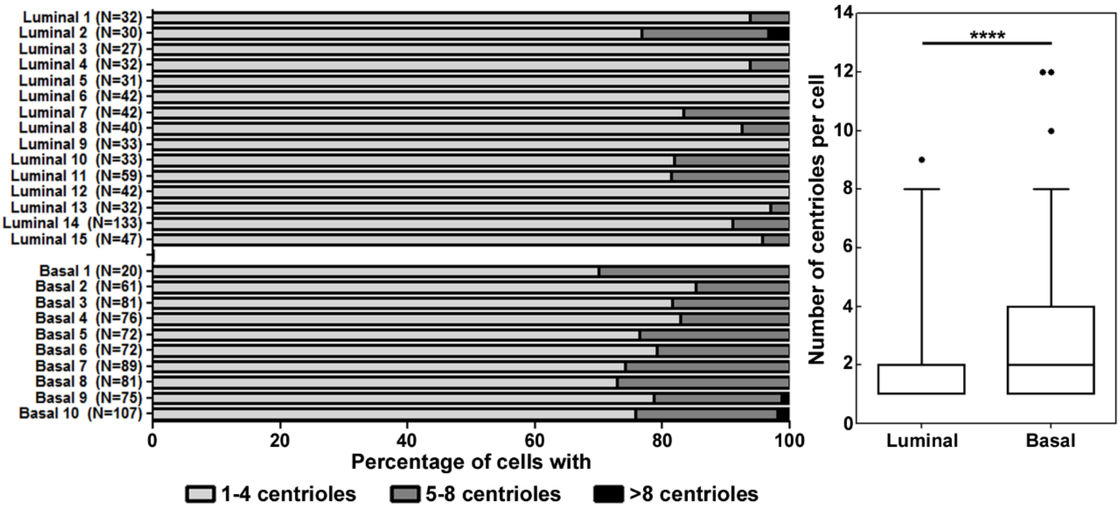

Supplementary Figure 6: Basal-like human breast carcinomas display higher levels of centriole amplification than luminal breast carcinomas. A series of formalin-fixed and paraffin-embedded human breast tumour samples, including 15 luminal carcinomas and 10 basal-like tumours, was analysed in order to identify and count centrioles by immunofluorescence microscopy. Tissue sections were stained with DAPI (blue), pericentrin (green) and GT335 (red) antibodies. Please note that only the GT335 dots positive for pericentrin were considered as *bona fide* centrioles to avoid false positives. **a** Table mentioning the total number, the percentage of cells with 1-4, 5-8 and >8 centrioles, the number of patients and the range of cells analysed for both luminal and basal-like tumours. Note that 8% and 22% of the cells display centriole amplification in luminal and in basal-like breast carcinomas, respectively. **b** Graph bars showing the percentage of cells with 1-4, 5-8 and >8 centrioles for each patient of the luminal and basal-like breast carcinomas. Note that cells with 5-8 centrioles were more commonly observed than cells with >8 centrioles. N represents the number of cells analysed per patient. **c** Box plot representing the distribution of the number of centrioles per cell observed in the luminal and basal-like tumours. Please note that the mean number of centrioles identified per cell was higher in basal-like carcinomas (2.88 ± 1.96) than in luminal tumours (2.09 ± 1.44) Whiskers: 1-99 percentile, $p<0.0001$ Mann-Whitney test.

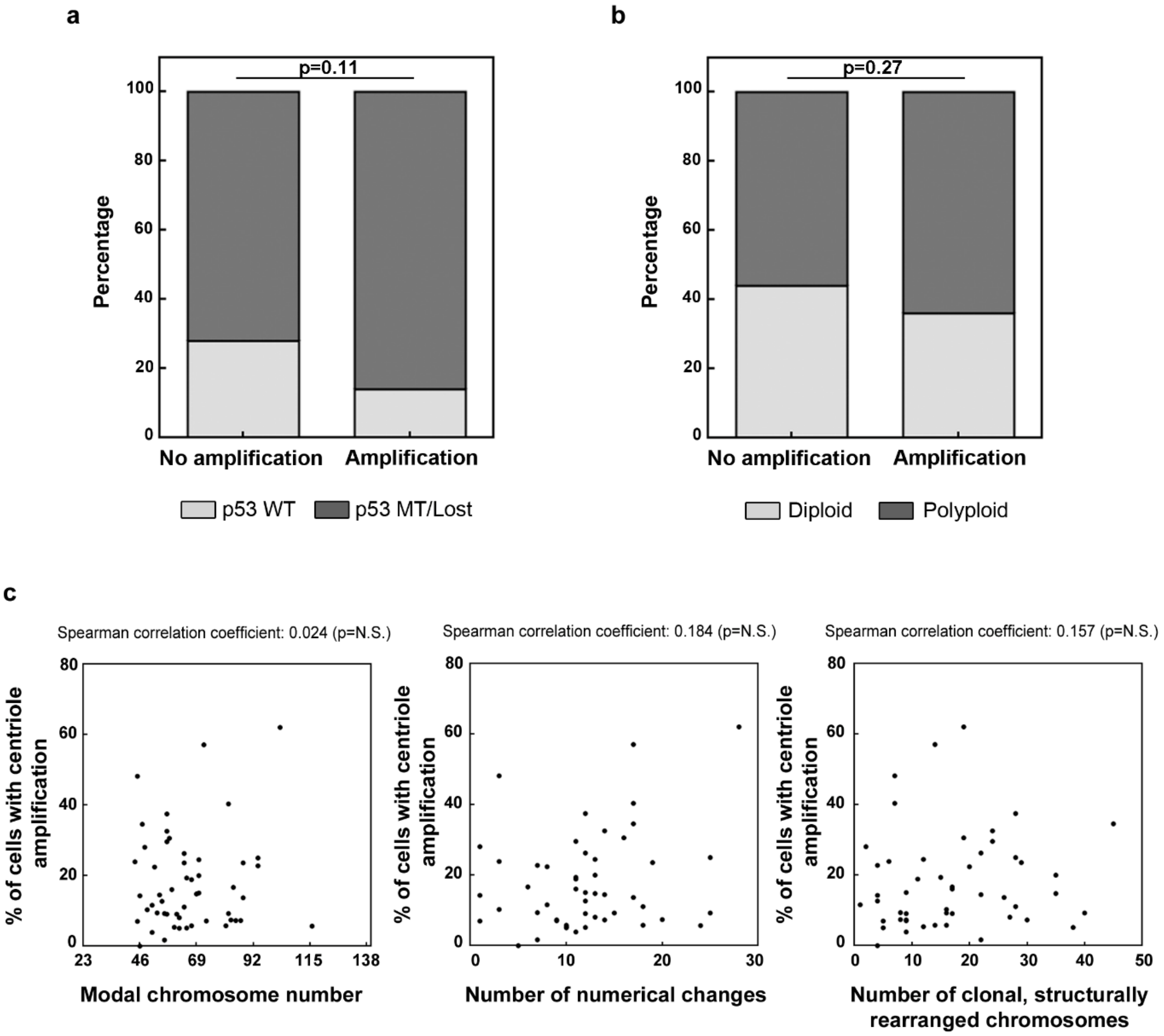

Supplementary Figure 7: Centriole amplification, loss of p53 function and ploidy deregulation. **a** The percentage of cell lines with loss of p53 function (p53 mutated (MT)/Lost - dark grey bar) or with intact p53 function (p53 Wild Type (WT) - light grey bar) is depicted in the group without (i.e. not significantly different from the non-cancerous cell lines) centriole amplification and in the group with significant centriole amplification. The proportion of p53 MT cell lines is higher in the group with significant amplification (86%) than in the low amplification group (72%) but these proportions are not statistically different (z score test, one-tailed, p=0.11). **b** The percentage of cell lines with increase in ploidy (polyploid, dark grey bar) or with normal ploidy (diploid - light grey bar) is depicted in the group without significant centriole amplification and in the group with centriole amplification. The proportion of polypoid cell lines in the group with amplification (64%) and in the low amplification group (56%) is not different (z score test, one-tailed, p=0.27, N.S.: not significant). The p53 and ploidy status were obtained from [2] and [3], respectively. **c** No Correlation is observed between the penetrance of centriole amplification and the modal chromosome number, the number of numerical chromosome changes and the number of structural chromosomal rearrangement observed in each of the cancer cell lines. For each of the analysed cell lines, the percentage of cells with > 4 centrioles (X axis) was plotted against the modal chromosome number, the number of numerical chromosome changes and the number of clonal structural chromosomal rearrangement obtained from [4].

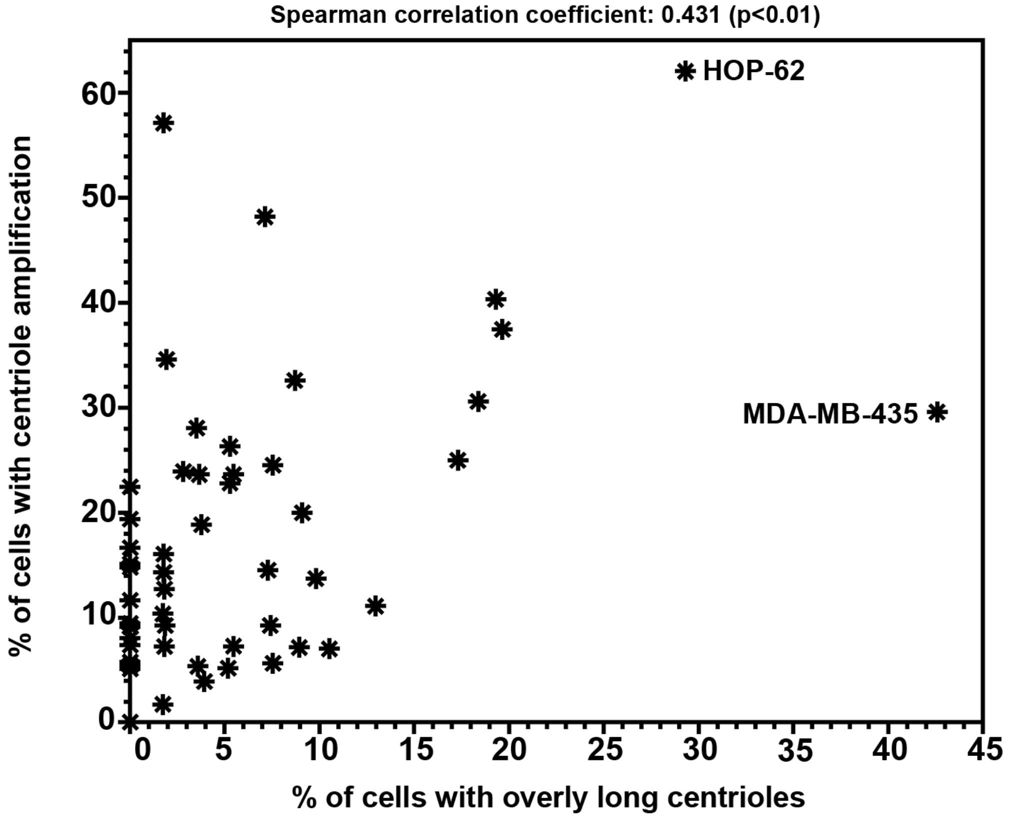

Supplementary Figure 8: Correlation between centriole elongation and amplification in cancer cell lines. For each of the analysed cell lines, the percentage of cells with overly-long centrioles (X axis) was plotted against the percentage of cells with > 4 centrioles (Y axis).

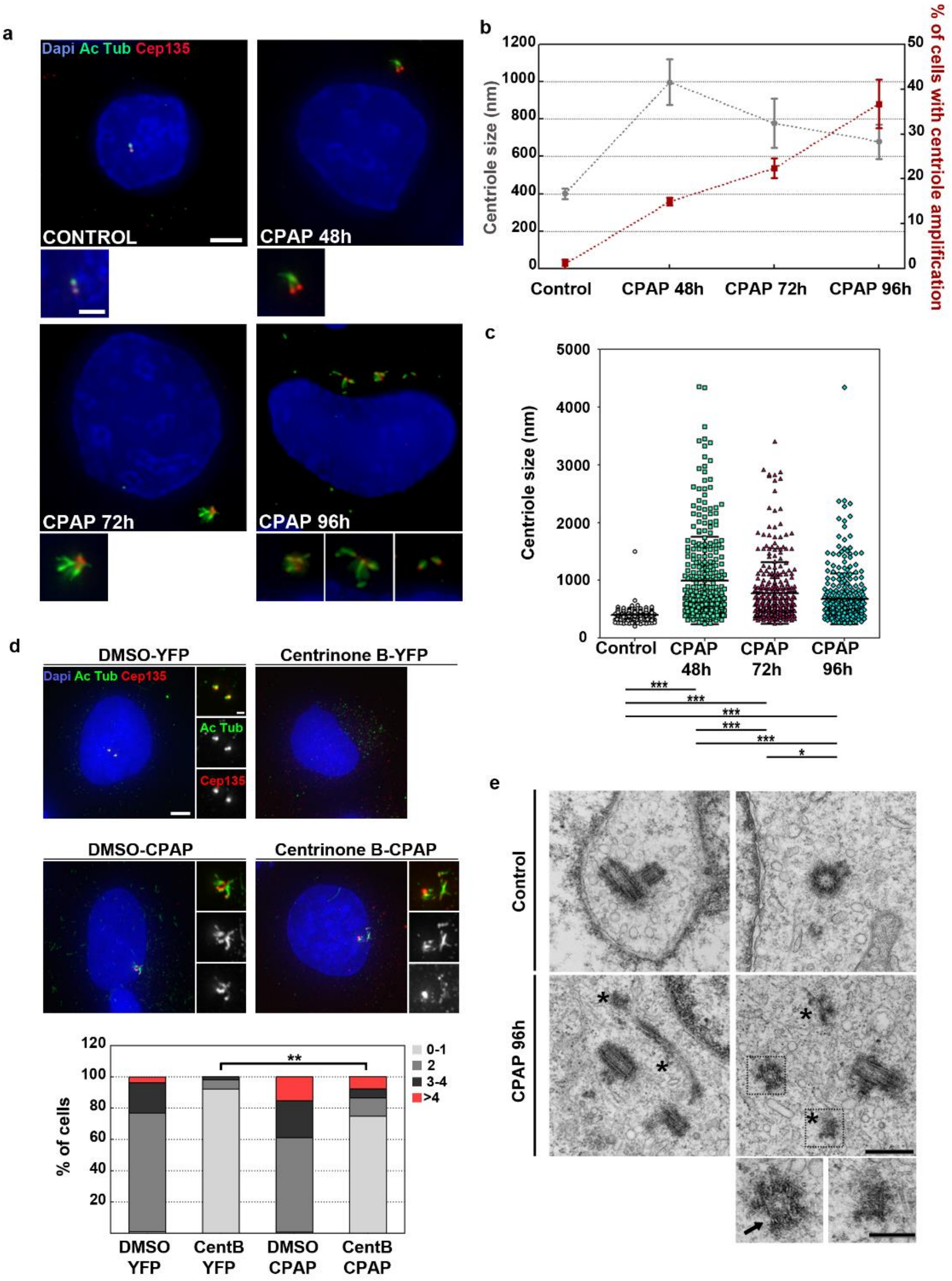

Supplementary Figure 9: Centriole over-elongation triggers amplification partially through centriole fragmentation. **a-c** CPAP was overexpressed in the osteosarcoma U2OS cell line (control – YFP expression). Cells were stained with DAPI (blue), acetylated tubulin (labelling stable MTs, green) and Cep135 (a proximal-end marker of centrioles, red) antibodies. **a** Representative Immunofluorescence images. Scale bar 4 µm, insets 2 µm **b** Graph representing centriole size (grey) and the percentage of cells with centriole amplification (>4, red), (for more details, see methods). Note that a significant increase in centriole length was observed upon CPAP overexpression (mean centriole length equal to 998 ± 122, 776 ± 132, 678 ± 92 nm at 48 h, 72 h and 96 h, respectively). Note the steady increase in cells containing >4 centrioles upon CPAP overexpression (15, 22 and 37 % at 24 h, 48 h and 72 h, respectively). Data represent the mean +/- s.d. of three independent experiments (100 centrioles measured and 100 cells counted per condition, and per experiment). **c** Raw data of the centriole size measurement of **b**. Bars represent the mean and error bars are defined as +/- s.d. * $p<0.05$, ** $p<0.01$ and *** $p<0.001$ (Mann- Whitney test). **d** Similar experiments to **a**-**c** were performed, except that cells were incubated with centrinone B or with DMSO (96 h of CPAP overexpression). Cells were blindly selected based only on the DAPI and not on the GFP channel as cells lacking centrioles would only have a faint cytosolic signal, therefore not being accounted for. While reducing experimental bias, this approach diminished the penetrance of centriole amplification in the DMSO-CPAP sample (around 15% instead of the 35% usually observed, see **b**), as non-transfected cells were counted. Scale bar 5 µm, insets 1 µm. Chi-square test, $p=0.003$. **e** CPAP overexpression promotes centriole structure alterations and fragmentation in U2OS cells. TEM pictures of centrioles in control (YFP) and in CPAP overexpressing cells. Defective centrioles lacking a microtubule doublet (arrow) and centriolar fragments (asterisks) were only present in cells overexpressing CPAP. Microtubules emanating from the centriole fragment are visible (lower inset), suggesting that centriolar fragments are functional MTOCs. Scale bar 500 nm, insets 250 nm.

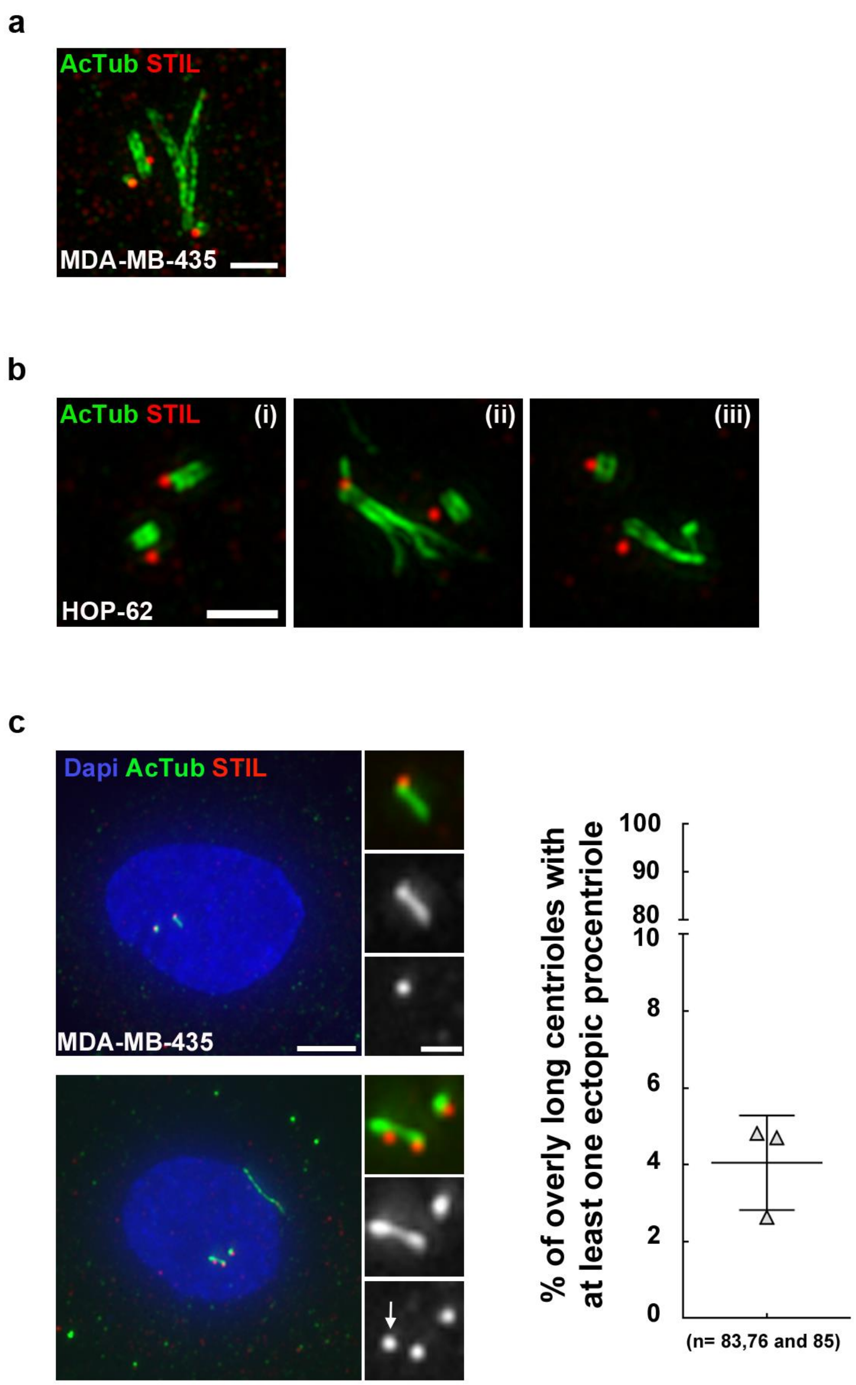

Supplementary Figure 10: Centriole over-elongation drives centriole amplification in cancer cells via ectopic procentriole formation and centriole fragmentation. **a** Structured Illumination Microscopy (SIM) picture showing a branched overly-long centriole in MDA-MB-435 cell line. Cells were subjected to a 2 h cold treatment, prior to fixation, to depolymerise the cytoplasmic MTs, and subsequently stained with DAPI (blue), acetylated-tubulin antibody (green) and STIL antibody (red). Scale bar: 1 µm. **b** Examples of Structured Illumination Microscopy (SIM) pictures showing normal-length centrioles (i), overly long centrioles with defective structures (ii) and a centriolar fragment (iii) in HOP-62 cell line. Cells were subjected to a 2 h cold treatment, prior to fixation, to depolymerise the cytoplasmic MTs, and subsequently stained with DAPI (blue), acetylated-tubulin antibody (green) and STIL antibody (red). Scale bar: 1 µm. **c** Quantification of ectopic procentriole formation in MDA-MB-435 cell line. Overly-long centrioles with at least two STIL dots along their length were accounted (see white arrow in lower panel). Cells were subjected to a 2 h cold treatment, prior to fixation, to depolymerise the cytoplasmic MTs, and were stained with DAPI (blue), acetylated-tubulin (green) and STIL (red) antibodies Bars represent the mean +/- s.d. of three independent experiments (83, 76 and 85 overly long centrioles were analysed). Scale bar: 5 µm, insets: 1 µm.

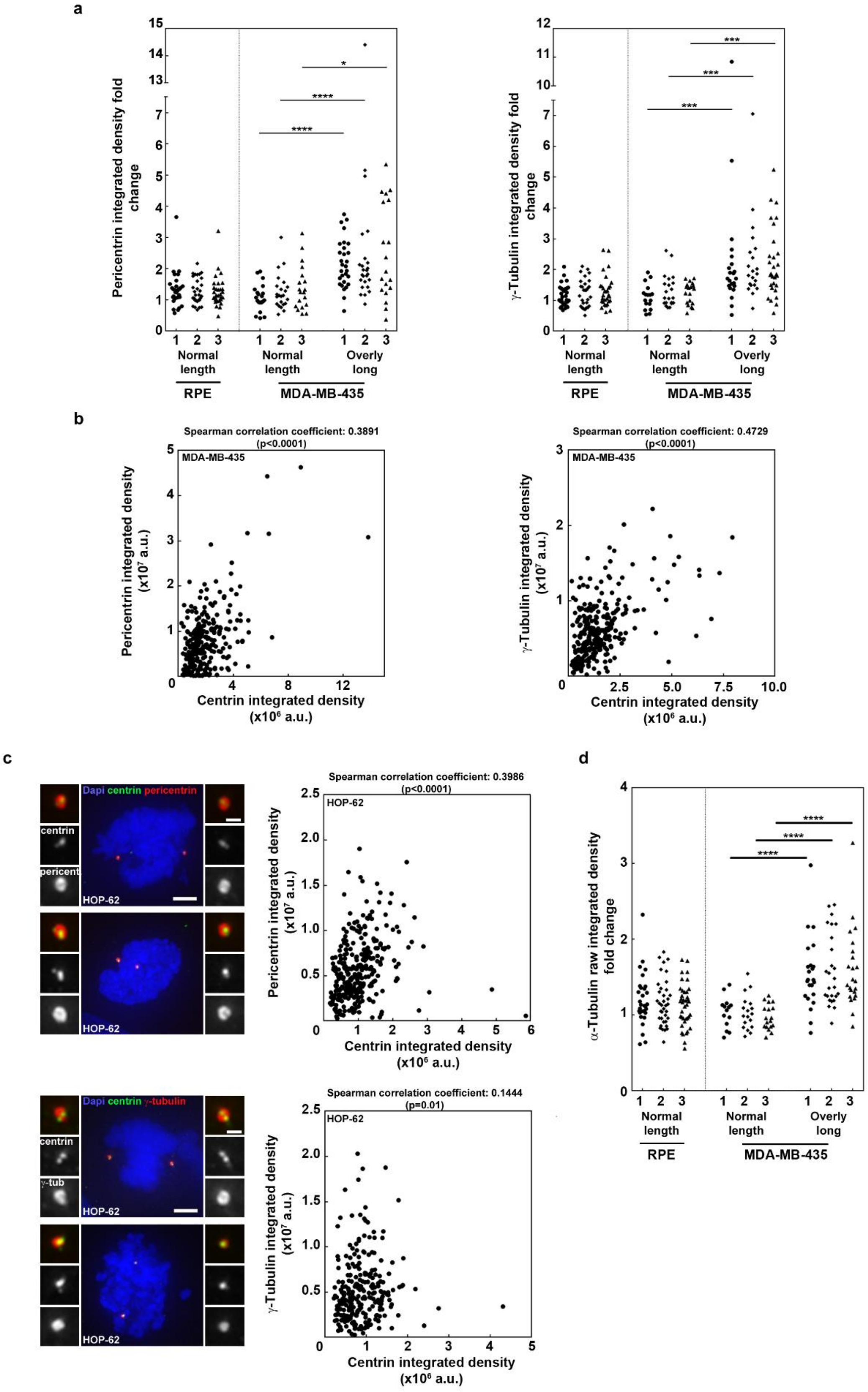

Supplementary Figure 11: The presence of overly long centrioles leads to the formation of over-active centrosomes. **a-c** Elongated centrioles recruit more pericentriolar material than normal-length centrioles. **a** Graph showing the fold changes in integrated density of pericentrin or γ-tubulin between mitotic poles of RPE-1 and MDA-MB-435 cells within each experiment (for more details on the quantifications, see methods section). Three independent experiments were performed (labelled 1, 2 and 3; between 37-62 centrosomes were quantified per

condition and per experiment). * $p \leq 0.05$, *** $p \leq 0.001$ and **** $p \leq 0.0001$ (Mann-Whitney test). **b** Positive correlation between centrin and PCM (pericentrin or γ-tubulin) integrated densities of MDA-MB-435 centrosomes. For each of the analysed centrosomes of **a**, the centrin integrated density (X axis) was plotted against the pericentrin or γ-tubulin integrated density (Y axis). A total of 272 centrosomes for pericentrin and 294 for γ-tubulin are plotted. **c** Positive correlation between centrin and PCM (pericentrin or γ-tubulin) integrated densities of HOP-62 centrosomes. Representative immunofluorescence images of another cell line from the NCI-60 panel that displays overly long centrioles, HOP-62 cell line. Cells were stained with DAPI for the DNA, with centrin (green), and γ-tubulin or pericentrin antibodies (two PCM components, red). The centrin integrated density (X axis) was plotted against the pericentrin or γ-tubulin integrated density (Y axis). A total of 305 centrosomes for pericentrin and 258 for γ-tubulin are plotted (three independent experiments were performed). Scale bar: 5 µm, insets: 1 µm. **d** Overly long centrioles nucleate more MTs than normal-length centrioles. Graph showing the fold changes in raw integrated density of α-tubulin between mitotic poles within each experiment (for more details on the quantifications, see methods section). Three independent experiments were performed (labelled 1, 2 and 3; between 30-82 centrosomes were accounted per condition and per experiment). **** $p \leq 0.0001$ (Mann-Whitney test).

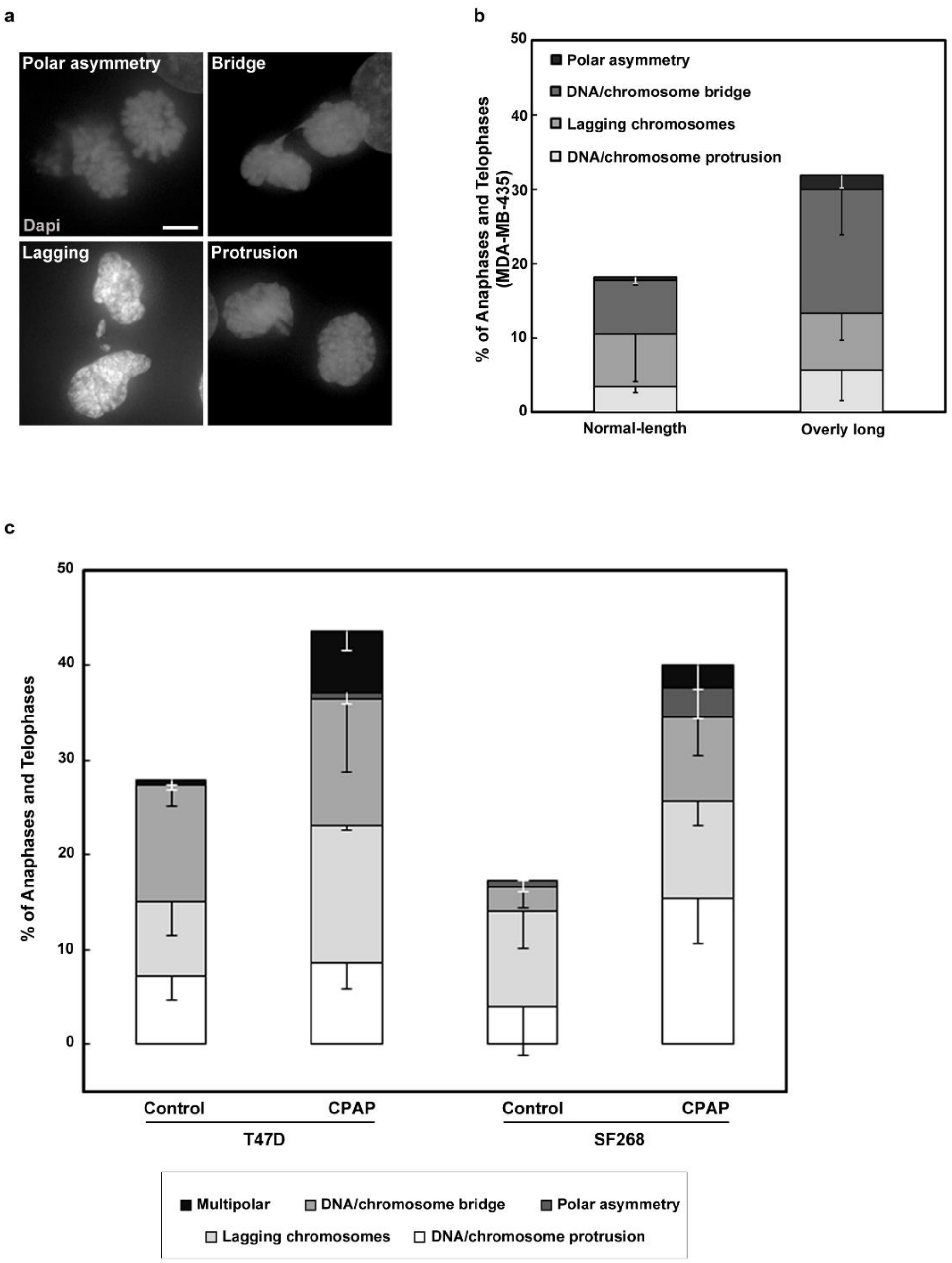

Supplementary Figure 12: Chromosome segregation defects are increased in mitotic cells with overly long centrioles. **a** Representative immunofluorescence images showing the different types of chromosome segregation defects scored in cells in anaphase and telophase stages: polar asymmetry (chromosome(s) in the vicinity of one of the two spindle poles at anaphase onset), DNA/chromosome bridge ("string-like" connection between the two masses of segregating chromosomes), lagging chromosome (chromosomes that lag between the two masses of segregating chromosomes) and DNA/chromosome protrusion (improperly aligned chromosome/DNA that probably results from bridges). For simplicity, only DAPI is represented here but MDA-MB-435 cells were also stained for centrioles and centromeres using centrin (green), CP110 (red), and CENPB antibodies (green), respectively. Scale bar 5 µm. **b** Graph showing the percentage of anaphases and telophases displaying the different aforementioned categories of chromosome segregation defects for MDA-MB-435 cells, containing, or not, overly long centrioles. 200 cells were analysed per experiment (n=3). The bars represent the mean - s.d. for each type of chromosome segregation defects. **c** Graph showing the percentage of anaphases and telophases cells displaying the different aforementioned categories of chromosome segregation defects plus multipolar spindle for T47D and SF268 cells, upon or not CPAP overexpression. Three independent experiments were performed (between 38-55 mitosis were accounted per condition and per experiment). The bars represent the mean - s.d. for each type of chromosome segregation defects.

Supplementary Table 1: List of the cancer cell lines of the NCI-60 panel and their respective tissue of origin, growth media and origin

Cell Line	Tissue of origin	Media	Origin
**MCF7**	Breast	DMEM + 10%FBS+P/S	Polyak lab (Dana-Farber Cancer Institute)
**T47D**	Breast	RPMI-1640 + 10%FBS + P/S	Polyak lab
**MDA-MB-231**	Breast	McCoy's +10%FBS+ P/S	Polyak Lab
**MDA-MB-468**	Breast	DMEM + 10%FBS + P/S	J.Paredes' lab (Ipatimup)
**BT-549**	Breast	RPMI-1640 + 10%FBS + P/S	ATCC
**HS578T**	Breast	DMEM + 10%FBS+P/S + 10ug/ml insulin	Polyak lab
**SF-268**	CNS	RPMI-1640 + 10%FBS + P/S	J. Barretina (The Broad Institute of Harvard and MIT)
**SF-295**	CNS	RPMI-1640 + 10%FBS + P/S	J. Barretina
**SNB-19**	CNS	RPMI-1640 + 10%FBS + P/S	J. Barretina
**SNB-75**	CNS	RPMI-1640 + 10%FBS + P/S	J. Barretina/D.Hancock (The Francis Crick institute)
**U251**	CNS	RPMI-1640 + 10%FBS + P/S	J. Barretina
**SF-539**	CNS	RPMI-1640 + 10%FBS + P/S	J. Barretina
**KM12**	Colon	RPMI-1640 + 10%FBS + P/S	J. Barretina
**HCT-15**	Colon	RPMI-1640 + 10%FBS + P/S	ATCC
**HT29**	Colon	DMEM + 10%FBS+P/S	Hanh's lab (Dana-Farber Cancer Institute)
**HCT-116**	Colon	DMEM + 10%FBS+P/S	ATCC
**SW-620**	Colon	Leibovitz's L-15 Medium + 10% FBS + P/S	ATCC/M. Godinho's lab (IGC)
**HCC-2998**	Colon	RPMI-1640 + 10%FBS + P/S	D.Hancock
**COLO205**	Colon	RPMI-1640 + 10%FBS + P/S	NOVARTIS
**SR**	Blood	RPMI-1640 + 10%FBS + P/S	ATCC
**K-562**	Blood	DMEM + 10%FBS+P/S	NOVARTIS
**MOLT-4**	Blood	RPMI-1640 + 10%FBS + P/S	J. Barretina
**RPMI-8226**	Blood	RPMI-1640 + 10%FBS + P/S	Pellman's Lab (Dana-Farber Cancer Institute)
**CCRF-CEM**	Blood	RPMI-1640 + 10%FBS + P/S	NOVARTIS
**HL-60**	Blood	RPMI-1640 + 10%FBS + P/S	J. Barrata's lab
**LOXIMVI**	Skin	RPMI-1640 + 10%FBS + P/S	J. Barretina
**SK-MEL-5**	Skin	RPMI-1640 + 10%FBS + P/S	ATCC
**M14**	Skin	DMEM + 10%FBS+P/S	Fisher lab (Dana-Farber Cancer Institute)/D.Hancock
**UACC-62**	Skin	RPMI-1640 + 10%FBS + P/S	D.Hancock
**SK-MEL-2**	Skin	RPMI-1640 + 10%FBS + P/S	J. Barretina
**SK-MEL-28**	Skin	RPMI-1640 + 10%FBS + P/S	ATCC
**UACC-257**	Skin	RPMI-1640 + 10%FBS + P/S	Fisher lab
**MDA-MB-435**	Skin	DMEM + 10%FBS+P/S	Polyak lab
**MALME-3M**	Skin	Iscove's Modified Dulbecco's Medium + 20% FBS + P/S	Pellman's Lab
**NCI-H226**	Lung	RPMI-1640 + 10%FBS + P/S	ATCC
**EKVX**	Lung	RPMI-1640 + 10%FBS + P/S	J. Barretina
**NCI-H522**	Lung	RPMI-1640 + 10%FBS + P/S	ATCC/D.Hancock
**A549**	Lung	DMEM + 10%FBS+P/S	ATCC
**NCI-H460**	Lung	RPMI-1640 + 10%FBS + P/S	ATCC
**HOP-92**	Lung	RPMI-1640 + 10%FBS + P/S	J. Barretina
**NCI-H322M**	Lung	RPMI-1640 + 10%FBS + P/S	J. Barretina
**NCI-H23**	Lung	RPMI-1640 + 10%FBS + P/S	ATCC/D.Hancock
**HOP-62**	Lung	DMEM + 10%FBS+P/S	D.Hancock

Cell Line	Tissue of origin	Media	Origin
**OVCAR-5**	Ovaries	1:1 mix of 199 and MCDB105 +10%FBS + P/S	Brugge lab (Dept of cell biology, HMS)
**NCI-ADR-RES**	Ovaries	RPMI-1640 + 10%FBS + P/S	J. Barretina
**IGROV1**	Ovaries	RPMI-1640 + 10%FBS + P/S	NOVARTIS
**OVCAR-8**	Ovaries	RPMI-1640 + 10%FBS + P/S	NOVARTIS
**OVCAR-3**	Ovaries	RPMI +20%FBS + P/S + 0,01 mg/ml insulin	Brugge lab
**SK-OV-3**	Ovaries	McCoy's +10%FBS+ P/S	Brugge lab
**OVCAR-4**	Ovaries	RPMI-1640 + 10%FBS + P/S	D.Hancock
**PC-3**	Prostate	F12K + 10%FBS + P/S	ATCC
**DU-145**	Prostate	Eagles + 10%FBS + P/S	NOVARTIS
**A498**	Kidney	Eagles + 10%FBS + P/S	ATCC
**UO-31**	Kidney	RPMI-1640 + 10%FBS + P/S	J. Barretina
**TK-10**	Kidney	RPMI-1640 + 10%FBS + P/S	J. Barretina
**786-0**	Kidney	RPMI-1640 + 10%FBS + P/S	J. Barretina/IPATIMUP
**SN12C**	Kidney	RPMI-1640 + 10%FBS + P/S	J. Barretina
**ACHN**	Kidney	Eagles + 10%FBS + P/S	NOVARTIS
**CAKI-1**	Kidney	McCoy's +10%FBS+ P/S	NOVARTIS
**RXF-393**	Kidney	RPMI-1640 + 10%FBS + P/S	D.Hancock

Please note that the origin of some cell lines differs between the primary and the secondary screening. We first mentioned the origin for the primary screening and then the origin for the secondary screening. The first time a lab appears, the host Institution is also mentioned.

Supplementary Table 2: Output of the primary and secondary screenings for centriole number and length, p53 and ploidy status of the NCI-60 cell lines.

Cell Line	Tissue of origin	% of cells with >4 centrioles PRIMARY SCREENING	% of cells with >4 centrioles SECONDARY SCREENING	% of cells with overly long centrioles PRIMARY SCREENING	% of cells with overly long centrioles SECONDARY SCREENING	p53 status	Ploidy	Modal chromo-some number	Number of clonal, structurally rearranged chromosomes	Number of numerical changes
MCF7	Breast	20.4	5.2	0.0	5.2	WT	3n-	65	38	12
T47D	Breast	16.1	9.1	3.6	0.0	MT	2n+	57	17	12
MDA-MB-231	Breast	73.6	14.5	28.3	7.3	MT	2n+	54	22	14
MDA-MB-468	Breast	20.4	23.6	7.4	3.6	MT	3n-	64	ND	ND
BT-549	Breast	49.1	24.5	23.6	7.5	MT	3n+/-	70	12	13
HS578T	Breast	45.1	32.6	9.8	8.7	MT	2n+	57	24	14
SF-268	CNS	9.4	1.7	1.9	1.7	MT	2n+	56	22	7
SF-295	CNS	29.6	5.7	3.7	7.5	MT	5n+/-	116	14	24
SNB-19	CNS	27.8	9.1	13.0	0.0	MT	3n+/-	61	9	12
SNB-75	CNS	37.7	12.7	5.7	1.8	MT	2n+	55	4	12
U251	CNS	31.4	22.4	7.8	0.0	MT	2n+	52	20	8
SF-539	CNS	17.0	23.6	3.8	5.5	MT	4n+/-	88	29	19
KM12	Colon	9.6	ND	0.0	ND	MT	2n+/-	43	7	8
HCT-15	Colon	12.1	ND	0.0	ND	MT	2n+/-	44	4	0
HT29	Colon	28.4	5.8	1.5	0.0	MT	3n+/-	67	16	10
HCT-116	Colon	35.8	7.0	17.0	10.5	WT	2n-	45	5	1
SW-620	Colon	48.1	10.3	11.1	1.7	MT	2n+/-	49	16	3
HCC-2998	Colon	18.2	23.9	0.0	2.8	MT	2n+/-	44	6	3
COLO205	Colon	50.0	57.1	9.6	1.8	MT	3n	72	14	17
SR	Blood	9.7	14.3	37.1	1.8	WT	2n+/-	46	4	1
K-562	Blood	17.9	19.4	12.5	0.0	MT	3n-	65	15	11
MOLT-4	Blood	70.9	22.8	5.5	5.3	MT	4n	94	4	7
RPMI-8226	Blood	29.1	26.3	1.8	5.3	MT	3n-	64	22	12
CCRF-CEM	Blood	21.8	28.1	3.6	3.5	MT	2n+/-	48	2	1
HL-60	Blood	28.8	48.2	5.1	7.1	MT	2n+/-	45	7	3
LOXIMVI	Skin	14.0	ND	2.0	ND	WT	3n+/-	64	11	12
SK-MEL-5	Skin	14.8	ND	0.0	ND	WT	4n+	100	16	27
M14	Skin	37.0	5.4	9.3	3.6	MT	3n+/-	60	12	10
UACC-62	Skin	11.5	7.1	7.7	8.9	WT	3n+/-	73	9	9
SK-MEL-2	Skin	60.0	9.3	8.0	7.4	MT	4n-	82	16	25
SK-MEL-28	Skin	73.1	13.7	30.8	9.8	MT	4n-	88	26	17
UACC-257	Skin	30.8	15.1	1.9	0.0	WT	3n+	70	9	12
MDA-MB-435	Skin	55.8	29.6	21.2	42.6	MT	2n+	57	24	11
MALME-3M	Skin	67.9	40.4	19.6	19.3	WT	4n+/-	82	7	17

Cell Line	Tissue of origin	% of cells with >4 centioles PRIMARY SCREENING	% of cells with >4 centioles SECONDARY SCREENING	% of cells with overly long centrioles PRIMARY SCREENING	% of cells with overly long centrioles SECONDARY SCREENING	p53 status	Ploidy	Modal chromo-some number	Number of clonal, structurally rearranged chromosomes	Number of numerical changes
**NCI-H226**	Non-Small Cell Lung	**10.0**	**ND**	**2.0**	**ND**	**MT**	**3n**	**62**	17	16
**NCI-H522**	Non-Small Cell Lung	**50.9**	**3.9**	**8.8**	**3.9**	**MT**	**2n+/-**	**51**	9	11
**EKVX**	Non-Small Cell Lung	**19.6**	**8.1**	**3.9**	**0.0**	**MT**	**3n+/-**	**62**	27	13
**A549**	Non-Small Cell Lung	**40.0**	**5.1**	**5.5**	**0.0**	**WT**	**3n+/-**	**62**	5	10
**NCI-H460**	Non-Small Cell Lung	**66.1**	**9.4**	**8.9**	**0.0**	**WT**	**2n+/-**	**53**	8	7
**HOP-92**	Non-Small Cell Lung	**31.5**	**25.0**	**7.4**	**17.3**	**MT**	**4n+/-**	**94**	28	25
**NCI-H322M**	Non-Small Cell Lung	**40.8**	**34.6**	**0.0**	**1.9**	**MT**	**2n+/-**	**47**	45	17
**NCI-H23**	Non-Small Cell Lung	**67.3**	**37.5**	**19.2**	**19.6**	**MT**	**2n+**	**57**	28	12
**HOP-62**	Non-Small Cell Lung	**74.1**	**62.1**	**51.7**	**29.3**	**MT**	**4n+**	**103**	19	28
**OVCAR-5**	Ovarian	**15.7**	**ND**	**5.9**	**ND**	**WT**	**2n+**	**54**	15	11
**NCI-ADR-RES**	Ovarian	**7.1**	**5.5**	**1.8**	**0.0**	**MT**	**2n+/-**	**ND**	ND	ND
**IGROV1**	Ovarian	**5.6**	**7.3**	**1.9**	**5.5**	**MT**	**4n+/-**	**85**	9	9
**OVCAR-8**	Ovarian	**15.5**	**9.3**	**1.7**	**1.9**	**MT**	**2n+**	**56**	40	15
**OVCAR-3**	Ovarian	**13.2**	**14.8**	**3.8**	**0.0**	**MT**	**3n+/-**	**69**	35	13
**SK-OV-3**	Ovarian	**30.9**	**16.7**	**7.3**	**0.0**	**MT**	**4n+/-**	**84**	17	6
**OVCAR-4**	Ovarian	**16.1**	**20.0**	**5.4**	**9.1**	**MT**	**3n+/-**	**70**	35	13
**PC-3**	Prostate	**42.6**	**7.3**	**14.8**	**1.8**	**MT**	**4n**	**87**	30	14
**DU-145**	Prostate	**38.6**	**16.1**	**3.5**	**1.8**	**MT**	**3n+/-**	**59**	17	11
**A498**	Renal	**7.4**	**ND**	**0.0**	**ND**	**WT**	**3n**	**74**	14	18
**UO-31**	Renal	**14.5**	**0**	**1.8**	**0.0**	**WT**	**2n+/-**	**46**	4	5
**TK-10**	Renal	**20.4**	**5.8**	**4.1**	**0.0**	**MT**	**4n**	**81**	14	18
**786-0**	Renal	**30.9**	**7.4**	**7.3**	**0.0**	**MT**	**4n+/-**	**83**	8	20
**SN12C**	Renal	**90.7**	**11.1**	**31.5**	**13.0**	**MT**	**3n**	**64**	28	18
**ACHN**	Renal	**5.6**	**11.6**	**0.0**	**0.0**	**WT**	**2n+/-**	**51**	1	8
**CAKI-1**	Renal	**31.4**	**18.9**	**19.6**	**3.8**	**WT**	**3n**	**67**	11	11
**RXF-393**	Renal	**39.6**	**30.6**	**11.3**	**18.4**	**MT**	**3n+/-**	**58**	19	16

Values below and above the thresholds selected to separate cell lines displaying non-significant amplification, i.e 13%, or over-elongation, i.e. 5%, from the ones with significant amplification/over-elongation are depicted in green and red, respectively. ND: Not determined. WT: wild type. MT: mutated. 2n-: hypodiploid, 2n+/-: near diploid, 2n+: hyperdiploid, 3n-: hypotriploid, 3n: triploid, 3n+/-: near triploid, 3n+: hypertriploid, 4n-: hypotetraploid, 4n: tetraploid 4n+/-: near tetraploid, 4n+: hypertetraploid, 5n+/-: near pentaploid. The p53 and ploidy status, modal chromosome number, number of clonal, structurally rearranged chromosomes and number of numerical changes were obtained from [2],[3] and [4] , respectively.

SUPPLEMENTARY REFERENCES

1. Loffler, H., Fechter, A., Liu, F.Y., Poppelreuther, S. & Kramer, A. DNA damage-induced centrosome amplification occurs via excessive formation of centriolar satellites. *Oncogene* **32**, 2963-2972 (2013).

2. Leroy, B. *et al.* Analysis of TP53 mutation status in human cancer cell lines: a reassessment. *Human mutation* **35**, 756-765 (2014).

3. Cell Miner^{TM} Database Version:2.1 – Cell line information. http://discover.nci.nih.gov/cellminer/celllineMetadata.do.

4. Roschke, A.V. *et al.* Karyotypic complexity of the NCI-60 drug-screening panel. *Cancer research* **63**, 8634-8647 (2003).
